# The End-Organ Impairment in Liver Cirrhosis: Appointments for Critical Care

**DOI:** 10.1155/2012/539412

**Published:** 2012-05-16

**Authors:** Antonio Figueiredo, Francisco Romero-Bermejo, Rui Perdigoto, Paulo Marcelino

**Affiliations:** ^1^Liver Transplantation Centre, Hospital Curry Cabral, 1069-166 Lisbon, Portugal; ^2^Critical Care and Emergency CGU, Puerto Real University Hospital, 11510 Cadiz, Spain; ^3^CEDOC, Faculdade de Ciências Médicas, 1169-056 Lisbon, Portugal; ^4^Intensive Care Unit, Hospital Curry Cabral, 1069-166 Lisbon, Portugal

## Abstract

Liver cirrhosis (LC) can lead to a clinical state of liver failure, which can exacerbate through the course of the disease. New therapies aimed to control the diverse etiologies are now more effective, although the disease may result in advanced stages of liver failure, where liver transplantation (LT) remains the most effective treatment. The extended lifespan of these patients and the extended possibilities of liver support devices make their admission to an intensive care unit (ICU) more probable. In this paper the LC is approached from the point of view of the pathophysiological alterations present in LC patients previous to ICU admission, particularly cardiovascular, but also renal, coagulopathic, and encephalopathic. Infections and available liver detoxifications devices also deserve mentioning. We intend to contribute towards ICU physician readiness to the care for this particular type of patients, possibly in dedicated ICUs.

## 1. Introduction

Liver cirrhosis (LC) is a late stage of progressive hepatic fibrosis is characterized by distortion of the architecture and formation of regenerative nodules and different degrees of liver function impairment; these patients are prone to a variety of complications reducing life expectancy markedly [1].

The World Health Organization (WHO) indicates that 10% of the world population has chronic liver disease; this represents approximately 500 million people with 20 million people worldwide having liver cirrhosis and/or liver cancer. Two million people worldwide die each year from hepatic failure. In the United States the number of discharges with chronic liver disease or cirrhosis as the firs t-listed diagnosis totaled 112,000 patients in 2007 and 29,165 deaths (9.7 deaths/10000 population) [2].

Decompensate end-stage liver disease (ESLD), acute-on-chronic liver failure, and acute liver failure (ALF) are critical situations, which lead to hepatic encephalopathy and multiple organ failure (MOF) [3]. These critical situations may require admission and end-organ support in an intensive care unit (ICU). The rationales for patient admission are recovery of liver function and to provide an effective bridging to liver transplantation (LT), the most effective therapy for the failing liver.

Liver transplantation is hampered by the shortage of organ donors, resulting in a great incidence of patients with ALF who die while awaiting LT [4, 5]. The availability of liver support therapies may alter the future perspective of these patients, which can be admitted more often to the ICU. Several liver support devices are available and may be nonbiological (MARS, Gambro Inc.; Prometheus device, Fresenius Medical Care; SEPET, Selective Plasma Exchange Therapy from Arbios Systems, Inc.) or may have a biological component (bioartificial liver, BAL).

The authors intend to perform a comprehensive review of the physiopathology of the patient with LC, focusing on critical care in light of the new liver support therapies.

## 2. The Impact of Cirrhosis on Cardio-Circulatory System

Cardiovascular complications in the patient with LC are a major cause of perioperative mortality and graft loss [6].

 The main cardiovascular disorders of LC are portal hypertension and a permanent state of hyperdynamic circulation. The increased blood flow in the splanchnic bed exacerbates the portal hypertension and consequently increases the incidence of esophageal varices, variceal bleeding, and ascites. In the pathophysiology of ascites the peripheral vasodilatation contributes to decreasing the effective circulating volume, which is sensed by the kidney as hypovolemia, leading to salt and water retention. In more severe cases, this leads to severe renal vasoconstriction and a decline in renal function, which characterizes the hepatorenal syndrome (HRS).

The hallmark of this state of hyperdynamic circulation is an increase in heart rate (HR), cardiac output (CO), and left ventricular ejection fraction (LVEF) and a decrease in systemic vascular resistance (SVR), mean arterial pressure (MAP), and blood vessel contraction. Multiple pathophysiological mechanisms have been proposed to explain these conditions (neurogenic, humoral, and vascular dysregulation) and are summarized in Table 1 [7]. Although the etiology is remarkably different, this hemodynamic pattern resembles that of sepsis patients [8]. It has been reported that arterial vasodilatation activates the sympathetic nervous system and the renin-angiotensin-aldosterone system, resulting in a tachycardia response. Blood volume is decreased at a central level (heart, lungs, and great vessels) and increased in the periphery (mainly the splanchnic circulation) [9]. With the evolution of the illness, there are other precipitating factors of clinical worsening such as autonomic dysfunction, desensitization of myocardial beta-adrenergic receptors [10, 11], conditioning hyporesponsiveness to treatment with vasoactive, and nitration of cardiac proteins [12].

In an experimental model in rats with induced portal hypertension, reductions in myocardial contractility and beta-adrenergic response were observed and these findings were associated with a possible altered excitation-contraction coupling, decreased sarcolemma L-type calcium channel density, and reduced calcium in the sarcoplasmic reticulum.

Portal hypertension also induces portosystemic collaterals that drive gut-derived substances directly to systemic circulation without hepatic clearance. Nitric oxide (NO) is overproduced in LC and is increased in peripheral circulation, which results in peripheral vasodilatation and secondary to architectural and vasoactive humoral changes in the liver. There is an increase in vasoactive molecules such as angiotensin II, endothelin 1, and cysteinyl leukotriene associated with decreased intrahepatic NO production. The net result is a progressive increase in intrahepatic vascular resistance and portal hypertension [13].

 B-type natriuretic peptide (BNP) concentrations are higher in cirrhosis. Pimenta et al. in a study that included 83 patients hospitalized with decompensated cirrhosis observed that BNP level in cirrhosis reflects cardiac systolic function and is an independent predictor of medium-term survival in advanced cirrhosis. Median BNP level was 130.3 (65.2–363.3 pg/mL). BNP levels above the median were associated with an increased occurrence of death within 6 months of discharge [14, 15].

With regard to pulmonary circulation vascular dilatation occurs, as well as, intrapulmonary shunts and low pulmonary vascular resistance. The alveolar-capillary disequilibrium hypothesis states that pulmonary vascular dilatation and the intrapulmonary shunts in patients with LC are the leading contributors to hypoxemia in advanced liver disease. The low pulmonary vascular resistance leads to a reduction in the intrapulmonary transit time and subsequently a decreased oxygen diffusion across the dilated pulmonary vessels. Another pattern of intrapulmonary vascular dilation is characterized by localized dilatation of parts of the pulmonary vasculature and associated with large arteriovenous shunting, with poor response to oxygen supplementation [16–18].

## 3. Cirrhotic Cardiomyopathy

Although the cardiovascular impact of the LC is of common knowledge for almost 60 years [19], its true prevalence remains unknown since it is not observed in all patients with LC, only in those with more advanced disease [20]. The recognition of a distinctive non-alcohol-related cardiomyopathy in LC is recent. Cirrhotic cardiomyopathy (CCM) is the term used to describe a group of features indicative of abnormal cardiac performance in cirrhotic patients that seems to be an independent entity, different from ethanol- induced cardiomyopathy.

Little data is available on myocardial changes due to LC. The course of progressive damage to the cardiovascular system can be silent and precipitate after stress maneuvers, making both diagnosis and treatment, in most cases, late and difficult. Only scattered clinical studies have specifically studied the features of the CCM, this could justify why there are no widely accepted diagnostic criteria. A working definition was proposed in the World Congress of Gastroenterology in Montreal (Canada) in 2005 and is detailed in Table 2.

If the CCM is not diagnosed in a timely manner or is treated improperly, it can lead to heart failure [21]. Rates of pulmonary edema rise up to 50% and hemodynamically significant arrhythmias have been reported in 27% of patients in the perioperative phase of LT. Fouad et al. [22] studied 197 post-LT patients and found prevalence close to 50% of cardiac decompensation, being the main cause of death in these patients.

The histopathology of CCM is nonspecific. The autopsy findings include increased ventricular volumes and mass, hypertrophy of cardiomyocytes, interstitial and intracellular edema, and signs of cellular injury; also, the left ventricle (LV) is thickened and less compliant. These findings are more evident in patients with ascites [23], and they are more frequent in the LV than in the right ventricle [24].

One of the factors thought likely to participate in the pathogenesis of CCM is the increase in intra-abdominal pressure in cirrhotic patients. However, as CCM has been described in patients without ascites, it appears that no mechanical factors would cause progressive cardiac deterioration (nitric oxide, TNF*α*, bile acids, and beta-adrenergic receptor dysfunction).

**Figure 1:**
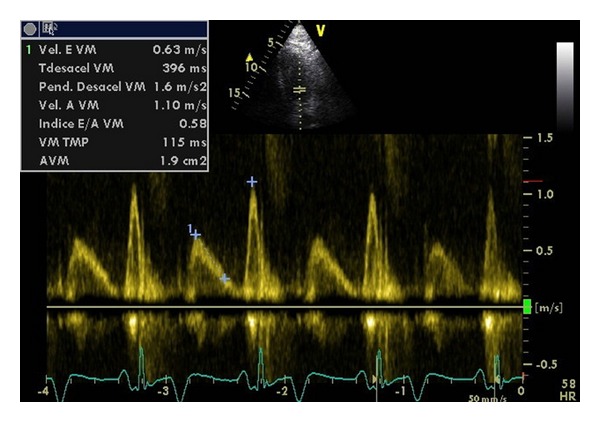
Diastolic dysfunction in a cirrhotic patient diagnosed during pretransplantation echocardiographic study (E/A ratio < 0.8, DTE > 240).

LV systolic function has been the most studied feature of CCM, which in particular is normal at rest but, in situations of physical stress (surgery, infection, and bleeding, exercise), psychological or pharmacological (dobutamine, sodium load) is affected [25]. In CCM the ejective period is shortened and preejective time is lengthened [26]. Diastolic dysfunction occurs due to a defect in ventricular compliance that alters its physiological filling, and yet not demonstrated in cirrhotic patients, some authors suggest its presence in all cases of CCM and the echocardiography finding of a pathological mitral E/A ratio may be sufficient for diagnosis (Figure 1) [27]. Nonetheless, it has been described in most cirrhotic patients without cardiomyopathy. If diastolic dysfunction is present, it will be more deteriorated after LT, especially in the first 3 months [28]. It appears to be more common in patients with ascites, and it is also demonstrated that paracentesis improves diastolic and systolic functions [29].

Electrophysiological changes have been described in patients with cirrhosis such as rhythm disturbances, QT prolongation, ventricular dyssynchrony, and chronotropic incompetence. Rhythm disturbances more frequently reported are atrial fibrillation, atrial flutter and extrasystoles; these may be due to changes in the permeability of the cell plasma membrane [30]. QTc prolongation is due to changes in myocardial repolarization and can lead to ventricular arrhythmias such as Torsade de Pointes, ventricular tachycardia, ventricular fibrillation, and even sudden death [31]. LT can improve the QTc interval of patients who have it prolonged [32].

Chronotropic incompetence is associated with increased risk of perioperative complications and can be defined as the failure to achieve 82% of the predicted heart rate after dobutamine echocardiography. It was associated with an increased risk of almost 4 times in the first months after LT (22% versus 6%) [33, 34].

Limited studies suggested that, in an earlier stage of CCM, mechanical dyssynchrony preceded LV dysfunction; in fact, mechanical dyssynchrony is one of the diagnostic criteria in the working definition of CCM. Recently, Aaroudi et al. [35] performed a study with 178 patients with LC who underwent stress-gated Tc-99m myocardial perfusion imaging and found no differences in dyssynchrony indexes between survivors and nonsurvivors, concluding that in patients with LC there is insufficient evidence of a higher incidence of LV dyssynchrony. 

The transjugular portosystemic shunt (TIPS) produces an acute increase in preload by shifting traffic from the portal vein circulation to the systemic circulation, leading to a worsening of the hyperdynamic state by increasing CO, biatrial end-diastolic volume, and a decrease in SVR. It is estimated that 1% of cirrhotic patients without cardiac history developed heart failure after TIPS [36]. With the TIPS, the central filling pressures increase more than 2 times. The stroke volume index increased up to 20% immediately [37]. Within two years, both CO and S VR showed a trend toward normalization, together with mild LV hypertrophy. These consequences are responsible for an increased probability of death in patients with CCM immediately after TIPS [38].

Another trigger for decompensation of CCM is LT. It is the third leading cause of death after rejection and infection [39], 47% of patients have radiographic acute pulmonary edema immediately after LT [40], and 3% developed new dilated cardiomyopathy in the first 6 months [41].

Several studies support the correlation between CCM and elevated atrial natriuretic peptide (ANP), BNP, pro-BNP, and troponin I, which could be used in screening. It seems that ANP is less specific since its alteration is related to atrial distension or distortion that can sometimes exist alongside effective hypovolemia [42]. Several studies have shown that when pro-BNP and BNP were elevated, these were related to the severity of cirrhosis, myocardial dysfunction, myocardial hypertrophy, and QT prolongation [43, 44]. Troponin I is a key parameter for the diagnosis of myocardial ischemia, which has proved to be useful for diagnosing other entities such as sepsis-induced myocardial dysfunction, hypertrophic cardiomyopathy, and LC. Pateron et al. [45] showed that patients with LC, who had elevated serum troponin I, correlated with lower LV stroke volume and mass index. Risk assessment with ECG, coronary angiography, and myocardial perfusion scintigraphy has failed to predict a perioperative CCM [46, 47].

The most common way to study the LV systolic function is using two-dimensional echocardiography. For the analysis of diastolic function, the mitral E/A ratio has been analyzed in most reported studies of CCM, concluding that a ratio ≤1 is associated with increased mortality risk. A mitral E/A ratio ≤1 is present in 50–70% of patients with ESLD, being more evident with the disease progression (MELD ≥ 20) and lowest in patients with ascites [48, 49]. However, the mitral E/A ratio is also preload dependent [50] and does not allow us to diagnose a pseudonormal pattern (grade II diastolic dysfunction), so the real incidence of diastolic dysfunction in patients with LC is underestimated. Recent studies using new echocardiographic technologies such as Doppler tissue imaging (DTI) are promising [51]. Kazankov et al. [52] studied LV systolic and diastolic functions using DTI at rest in 44 cirrhotic patients without previous known heart disease and simultaneously analyzed tissue velocities and strain rate in various segments of the LV in the same cineloop. They noted that all patients had systolic dysfunction and 54% had diastolic dysfunction (25% impaired diastolic relaxation pattern, 27% pseudonormal filling pattern, and 2% restrictive filling pattern) at rest. These findings suggest that the current characterization of the CCM is doubtful, further studies are needed in this field. Advanced methods such as echocardiography with DTI and speckle tracking may become the gold standard for CCM diagnosis.

Unfortunately there is no specific treatment for CCM [53]. Few treatments have been proposed to date and many of them are simply experimental. The clinical management of patients with CCM includes supportive measures.

Accumulating evidence suggests the use of cardioprotective drugs, such as beta-blockers, statins, angiotensin-converting enzyme inhibitors, or antialdosterone agents [54–58].

Albumin dialysis using the molecular adsorbent recirculating system (MARS) has been shown to improve hemodynamic status of patients with ESLD. Nonetheless, these effects disappeared four days after cessation of MARS. [59, 60].

## 4. Portopulmonary Hypertension

Portopulmonary hypertension (PoH) is usually defined using parameters obtained by right heart catheterization in patients with PoH. These include elevated pulmonary mean artery pressure (>25 mmHg at rest or >30 mmHg with exercise, or pulmonary artery systolic pressure >45 mmHg), increased pulmonary vascular resistance (PVR > 240 dynes s/cm^5^), normal pulmonary capillary wedged pressure (PCWP <15 mmHg), or transpulmonary gradient (the difference between mean pulmonary artery pressure, MPAP, and PCWP) >12 mmHg.

PoPH is a relatively rare condition, affecting 5% to 6% of LC patients [61] and commonly observed among patients with end-stage liver disease, biliary atresia, portal vein thrombosis, Budd-Chiari syndrome, and schistosomiasis.

Pathological and pathophysiological features include vascular alterations, such as intimal fibrosis, increased medial thickness, pulmonary arteriolar occlusion, in situ thrombosis, and plexiform lesions resulting from intramural endothelialization of microaneurysms; dysregulation of vascular, proliferative, and angiogenic mediators; hyperdynamic circulation leading to increased shear stress within pulmonary vasculature followed by a neurohumoral response characterized by elevated levels of endothelin-1 (ET-1); the excess of ET-1 acting through ET_A_ receptor, inducing pulmonary vasoconstriction, smooth muscle proliferation, and fibrosis; upregulation of other neurohumoral mediators (thromboxane and interleukin-6); decreased synthesis, in the pulmonary vasculature, of NO due to decreased levels of nitric oxide synthase [13, 14, 62].

Early stage of PoPH is generally asymptomatic. With advancing disease the dyspnea with exertion is the most frequent presenting symptom (81%); other symptoms, such as syncope, chest pain, and fatigue are seen in a third of the patients. PoPH can affect patients at any age and occasionally occur in noncirrhotic patients with PoH and have been also reported in mild liver disease. The disease staging is presented in Table 3.

A carefully evaluation must be made to exclude other potential causes of pulmonary hypertension (Table 4). Chest radiography may be normal in the early stages but in more advanced stages can show prominent main and central pulmonary vessels, sharply peripheral vessels, and right ventricle enlargement. Computed tomography chest scan shows an increased pulmonary artery diameter. Lung perfusion testing may also show multiple patchy defects. Arterial blood gases show a chronic respiratory alkalosis and impaired arterial oxygenation.

Transthoracic echocardiography using Doppler estimation of pulmonary artery systolic pressure is the screening tool of choice, using a threshold cut-off value of 30 mmHg; the sensitivity is 100% and the specificity is 96%.

**Figure 2:**
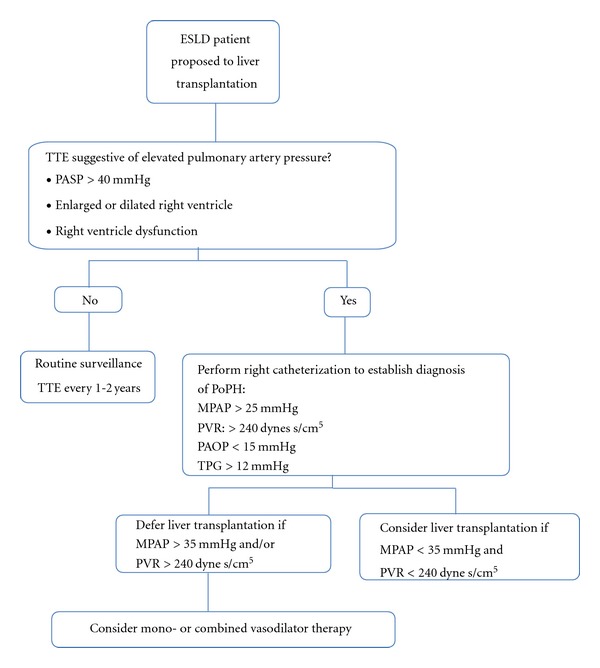
Screening and management of Portopulmonary hypertension (Modify of The University of California, San Francisco).

Regarding LC moderate to severe PoPH confers a high risk of cardiopulmonary-related mortality [63]; based on this many centers use pretransplantation evaluation of MPAP as shown in the Figure 2. Current treatment options are presented in Table 5.

## 5. Hepatopulmonary Syndrome

Clinical presentation is characterized by dyspnea of insidious onset associated with cyanosis in 90% of all cases, platypnea, and orthodeoxia. The presence of clubbing has the highest positive predictive value (75%) and dyspnea the highest negative predictive value (100%) for hepatopulmonary syndrome (HPS). Spider nevi are a common clinical feature of patients with HPS with a significant relationship between cutaneous spider angiomata and systemic and pulmonary vasodilatation suggesting that spider nevi may represent a cutaneous marker for intrapulmonary vascular dilatations. The main characteristics of HPS are intrapulmonary vascular vasodilatation, associated with significant arteriovenous shunting (AVS) and hypoxemia. Capillary vasodilatation is most pronounced at the lung bases, explaining orthodoxia and platypnea associated with HPS. V/Q (ventilation/perfusion) mismatch appears to be a major event in the pathogenesis of hypoxemia in HPS as a result of extensive pulmonary vasodilatation, a decrease in V/Q ratio in alveolar-capillary units, and resultant low PO_2_ and O_2_ content of blood leaving the lungs. The defect in oxygenation is due to pulmonary capillaries dilatation; oxygen encounters difficulty in diffusing into the centre of the larger capillaries. Increased CO and the associated reduced transition time of blood through the pulmonary vascular bed also impair diffusion, leading to a diffusion-perfusion defect or alveolar capillary oxygen disequilibrium.

Two types of HPS have been described: type I is associated with vascular dilatations at the precapillary level close to the normal gas exchange units of the lung; type II with focal larger dilatations amounting to arterial-venous shunting distant from the gas exchange units.

Supplementary oxygen improves type I HPS PaO_2_ but not type 2 HPS [13, 64]. The HPS is associated with an increased risk of death, and the median survival time in cirrhotic patients has been reported as 10.6 months compared to 40.8 months in cirrhotic patients without HPS. Survival is worse with baseline PaO_2_ <50 mmHg. The leading cause of death is hemorrhagic shock due to gastrointestinal bleeding [65].

The diagnosis relies on imaging techniques and arterial gas analysis. The cut-off values of PaO_2_ are controversial. Schenk and colleagues [66] suggested that arterial hypoxemia defined as a PaO_2_ <70 mmHg or below the age-related threshold predicted the presence of HPS with high probability in the absence of intrinsic cardiopulmonary diseases. A chest radiograph (CXR) and pulmonary function tests must be used to help exclude other causes of hypoxia such as pulmonary atelectasis, ascites, chronic obstructive pulmonary disease, and hepatic hydrothorax.

A definitive diagnosis of HPS can be made by demonstration of pulmonary vasodilatation associated with functional arteriovenous shunting. Imaging studies such as contrast echocardiography and perfusion scintigraphy with 99mTc can be used. A simple echocardiography method can also be used consisting in the injection of 10 mL of 0.9% saline; the normal time of appearance of this “contrast” on the left heart chambers is 4 heart beats; if the saline is visualized earlier, it means that a rapid transpulmonary circulation may be present and HPS is suspected [67].

Liver transplantation is the only definitive treatment for HPS with at least 85% of patients experiencing significant improvement or complete resolution of hypoxemia following surgery; however, these patients have a higher post-transplant mortality rate. Several therapeutic trials in HPS have shown poor results such as somatostatin analogues, cyclooxygenase inhibitors, and immunosuppressive agents namely corticosteroids and cyclophosphamide [68]. Some reports have shown improvement in gas exchange with the use of TIPS in HPS [69]. Martínez-Pallí et al. [70] in another study concluded that TIPS neither improved nor worsened pulmonary gas exchange in patients with portal hypertension.

## 6. Renal Dysfunction

Renal dysfunction in patients with advanced liver disease is characterized by increased renal sodium and solute-free water retention leading to ascites and hyponatremia, renal vasoconstriction with decreased glomerular filtration rate (GFR).

The diagnosis of renal failure in patients with cirrhosis is defined as an increase in serum creatinine 1.5 mg/dL, which corresponds to a GFR of 30 mL/min. Other definitions such as acute kidney injury (AKI) can be used. AKI is defined as an abrupt (two days) reduction in renal function manifested by an absolute increase in serum creatinine of at least 0.3 mg/dL or a percentage increase in serum creatinine of more than or equal to 50% (1.5 fold from baseline) or a urine output of less 0.5 mL/Kg/hour for more than six hours.

New biomarkers of renal function in cirrhosis are currently being studied as markers of renal disease in patients with advanced liver disease: neutrophil gelatinase-associated lipocalin (NGAL) and kidney injury molecule-1 (KIM-1). One study observed that plasma NGAL and APACHE II were the most powerful predictors of severe AKI within the first 2 days after LT [71].

It is important to remember that there are several causes of renal failure in cirrhosis: renal failure associated with infections, hypovolemia-induced renal failure, intrinsic renal disease, hepatorenal syndrome, and drug-induced renal failure.

One study, which included 562 hospitalized patients with cirrhosis and renal failure, found that renal failure was associated with bacterial infections in 46% of cases, hypovolemia-induced renal failure in 32%, hepatorenal syndrome in 13%, parenchymal nephropathy in 9%, and drug-induced renal failure in 7.5% [72]. 

Hyponatremia develops in the setting of ascites, and is associated with poor prognosis hepatic encephalopathy, especially when serum sodium levels is less than 130 mEq/L, due to changes in serum osmolality that lead to astrocyte swelling.

Some studies indicate that hyponatremia in post-LT patients is related to an increased risk of developing renal failure and bacterial infections during the first month after orthotopic liver transplantation, with a 3-month increased mortality [73, 74]. 

## 7. Hepatorenal Syndrome

The hepatorenal syndrome (HRS) refers to the development of renal failure in a patient who has advanced liver disease. Signs of portal hypertension, in particular ascites, must be present; without them, the diagnosis of HRS is compromised. Diagnostic criteria are presented in Table 6.

There are two types of HRS: type 1 is rapidly progressive with serum creatinine doubling to more than 2.5 mg/dL or a 50% reduction in creatinine clearance to less than 20 mL/min in a period of less than 2 weeks. Such patients may be classified as oliguric (defined as less than 500 mL of urine per day), but most are nonoliguric at the time of HRS diagnosis (if oliguria is defined as less than 400 mL per day). This form often occurs in an in-patient setting after a precipitating event, usually accompanied by concurrent bacterial infection, gastrointestinal bleeding, recent surgery, acute hepatitis and signs, and symptoms of severe hepatic insufficiency with jaundice, coagulopathy, encephalopathy, and circulatory dysfunction [75, 76].

Type 2 is notable for a steady and slow progressive rise in SCr in an out-patient setting in the cirrhotic patient with ascites. The overall survival rate is about 50% at 1 month and 20% at 6 months. Mortality is higher with type 1 HRS than with type 2 HRS, with a median survival of 2–4 weeks versus 5–6 months, respectively [76].

## 8. Coagulation Abnormalities

The hemostatic state in patients with severe liver disease may be considered as a state of reduced capability to maintain the fragile hemostatic balance. The liver is the site of fibrinogen factors II, V, VII, IX, XI, XII, and XIII synthesis. Also in the liver vitamin-K-dependent postribosomal conversion of glutamic acid residues in the protein precursor to gamma-carboxyglutamic acid takes place; this is an active process in the blood coagulation. A failure of carboxylation of coagulation factors results in an abnormal production of protein molecules that are nonfunctional [77].

Patients with liver disease and LC have a disturbed balance of procoagulant and anticoagulant factors characterized by an increased bleeding risk, decreased production of nonendothelial cell-derived coagulation factors, thrombocytopenia, altered platelet function, platelet inhibition by NO, abnormalities of fibrinogen, and decreased thrombin activatable fibrinolysis. In severe liver disease there is an increased concentration of tissue plasminogen activator due to its decreased hepatic clearance and the decreased thrombin activatable fibrinolysis inhibitor, as well as a decreased concentration of alpha-2-antiplasmin. There is also increased thrombotic risk due to decreased levels of liver-synthesized proteins C and S, alpha-2-macroglobulin, and antithrombin III. At the same time levels of plasminogen and heparin cofactor II are elevated.

Relative to thrombocytopenia and platelet dysfunction many theories have arise: decreased level of thrombopoietin, splenic sequestration of platelets due to portal hypertension, autoantibody destruction of platelets, and bone marrow suppression. Platelet dysfunction is also a contributing factor to decreased clot formation. In addition, patients with liver disease and concomitant renal insufficiency (such as different forms of hepatorenal syndrome) may have platelet dysfunction due to uremia and due to changes in vessel wall endothelial function. This is a common and often overlooked aspect of impaired hemostasis in cirrhosis [78].

## 9. Hepatic Encephalopathy Syndrome

In cirrhosis the onset of hepatic encephalopathy is related to precipitating factors that expose the brain to toxins. Hyperammonemia is a common feature that occurs due to the hydrolyzation of ammoniac molecules, which acquire hydrosolubility, facilitating the accumulation of these dangerous products in the organism.

The increased diffusion of ammonia into the brain is explained by a diminished hepatic clearance and increased blood-brain barrier permeability. There is an increased GABAergic tone induced by hyperammonemia, which produces behavioral disturbances. Parallel to this process the patients with hepatic encephalopathy show a decreased consumption of oxygen and glucose.

The ammonia could be related to the inhibition of cerebral energy metabolism, but it is not clear whether the decrease in oxygen consumption is the cause or the consequence of encephalopathy. A high lactate/pyruvate ratio was found in patients with acute liver failure indicating an impaired brain energy metabolism in the absence of hypoxia, an explanation for these findings could be the inhibition of limiting steps in the Krebs cycle and compromised oxidative phosphorylation in the mitochondrial inner membrane; an excessive glutamatergic activation leads to enhanced glycolysis with an increased lactate concentration. The West Haven criteria for hepatic encephalopathy are presented in Table 7.

The development of brain edema is related to the intensity and duration of hyperammonemia, as the increased plasma ammonia concentration leads to an increased lactate concentration that decreases brain pH and causes injury to the atrocities leading to the accumulation of water in the intracellular compartment. Hyponatremia may exacerbate astrocyte swelling due to differences in osmolality between the intracellular and the extracellular compartments. In cirrhosis, hyponatremia is associated with poor short-term prognosis [79]. The risk of edema is very low in grade 1 to 2 hepatic encephalopathy, and it increases in grade 3 to 25 (35%) and in grade 4 to 65 (75%).

The management of high-grade encephalopathy with brain edema and intracranial hypertension could be helped by using neuroimaging techniques and invasive monitoring of intracranial pressure (ICP). Supportive measures include: airmway protection; ventilation control and hyperventilation; preservation of adequate cerebral pressure; elevation of the head of the bed; and osmotic diuresis with mannitol. The treatment is aimed to reverse precipitating factors such as gastrointestinal bleeding, excess dietary protein, azotemia, constipation, hyponatremia, hypokalemia, metabolic disturbances, hypoxia, hypovolemia, infection, surgery, transjugular intrahepatic portosystemic shunt, narcotics, and sedative drugs.

The pharmacological therapy for hepatic encephalopathy is based on the control of ammonia production and uptake in the gastrointestinal tract and an increase in its elimination. For the reduction of intestinal ammonia production and absorption nonabsorbable disaccharides like lactulose and lactitol are the first line of therapy for hepatic encephalopathy.

Lactulose mechanism of action is based on its conversion to organic acid and the consequent acidification of the colon, which results in the suppression of ammoniagenic bacteria and the conversion of ammonia to ammonium causing a direct osmotic cathartic effect. The use of oral antibiotics is under major criticism and is summarized in Table 8.

## 10. The Cardiocirculatory Impact of Artificial Liver Support Systems

Two devices are commercially available in Europe, the Molecular Adsorption and Recirculating System (MARS), a technique of albumin dialysis, and fractionated plasma separation adsorption and dialysis (FPSA), Prometheus, a technique of detoxification. The global objective for these techniques is to support liver function during periods of disease exacerbation or acute or chronic liver disease. The clinical experience has shown that the benefits of these techniques are limited. Also, despite reports of better survival in patients waiting for LT [2], a systematic review of the few available, small, randomized trials, by the Cochrane Institute, concluded that overall liver support systems did not appear to affect mortality or effectively bridge patients to LT [80].

At this time there is greater knowledge on the possibilities of the artificial support systems. We know that they are effective in reducing the serum bilirubin levels, as well as, in selected patients, in improving encephalopathy. But several other problems must be mentioned: the propensity for coagulation abnormalities, a result from the use of nonfractionated heparin in patients with previously altered coagulation, and hemodynamic instability.

Few studies evaluated the hemodynamic impact of these devices. Laleman et al. [81] studied 18 patients and observed an increase in SVR resulting in an increase in MAP in patients submitted to MARS therapy but not in patients subjected to Prometheus, thus attenuating the typical hyperdynamic pattern. Also, in MARS patients a decrease in plasma rennin activity, aldosterone, vasopressin, and nitrate/nitrite levels was observed [82]. In our experience we also noticed that patients submitted to Prometheus are more prone to hypotension. Although this device does not alter the hyperdynamic profile, its extracorporeal circuit reaches 900 mL; in the MARS system, this extracorporeal circuit reaches only 150 mL. Also, Dethloff et al. [83] observed a decrease in platelet count with the use of Prometheus.

A modification in plasma levels of inflammatory cytokines was observed in patients subjected to MARS therapy [84]. There is no data on the long-term effect of these devices on the cardiocirculatory system.

An improvement in hepatic encephalopathy was observed by some authors [85]. However, despite these observations, a favourable impact on overall survival with the use of MARS and/or Prometheus is still lacking [86]. The experience on the use of liver support devices with biological components (bioartificial liver, BAL) is limited.

## 11. Infectious Problems in Patients with Liver Cirrhosis

Infections stand amongst the major causes for decompensating liver cirrhosis and contribute towards the mobility and mortality of these patients. Bacterial infections are present at admission or develop in approximately 30% of cirrhotic patients. About 60% of infections are community acquired and the last 40% are nosocomial. The most common infections are spontaneous bacterial peritonitis (SBP), urinary tract infections, pneumonia, and bacteremia without recognizable focus [87]. Bacteremia without recognizable focus is 10 times more frequent in liver cirrhosis patients, and up to 12% of liver cirrhosis patients may experience it [88, 89]. The most important bacterial agents are Gram-negative bacteria.

Patients with liver cirrhosis have immune dysfunction, making them more prone to infection. The recognized factors involve a decreased bactericidal function of plasma, decreased opsonization capacity, impaired macrophage FC*γ*-receptor-mediated clearance of antibody-coated bacteria, downregulation of HLA DR expression and lower complement levels, and functional alterations of the neutrophils [90, 91]. It is also associated with altered immune function and cytokine release in response to infection. Zimmerman et al. [92] showed that alcoholic patients exhibited lower plasma levels of interleukine 8 (IL8) during the onset of infection; in septic shock, alcoholic patients presented decreased levels of IL-1*β*, IL-6, and IL-8. Other anti-inflammatory cytokines, like IL-10, and proinflammatory TNF receptors did not differ from cross-matched patients without alcohol intake.

Data from the Cochrane database [93] showed that mortality due to SBP has improved in the last decade, but other infections did not, namely, spontaneous bacteriemia and pneumonia. LC patients are at high risk for the development of pulmonary infections [94]. Apart from immunological functions, there other important factors such as mechanical (reduced lung expansion due to the presence of ascites), altered mental status and reduced cough reflex, and intoxications. Also, the incidence of severe complications, such as the adult respiratory distress syndrome (ARDS), occurs more frequently in LC patients [95]. Viasus et al. [96] described a series of 90 LC patients with community-acquired pneumonia, where the most prevalent agents were *Streptococcus pneumonia* and *Pseudomonas aeruginosa*. These authors also described that the severity of liver dysfunction, accessed by the MELD score, was an independent risk factor for mortality.

Severe sepsis is also more common in cirrhosis patients. In its initial stages a cytokine storm converts responses that are usually beneficial into excessive damaging inflammation. Cirrhosis patients present a dual phenomenon: there is an imbalance in cytokine response and the decompensated liver disease is characterized by a pattern that closely resembles the sepsis-associated patterns. Then, the inflammatory phase is followed by a prolonged compensatory anti-inflammatory response syndrome responsible for nosocomial infections and increased death rate [90]. On the other hand patients with liver cirrhosis suffer from coagulation and liver function abnormalities, situations related or aggravated in severe sepsis; the vasopressor release is already dysfunctioning due to the underlying disease. These factors may be responsible for a more rapid and severe disease, contributing to higher mortality in the ICU. All pathologic alterations related to cirrhosis described earlier can effectively contribute to morbidity and mortality of these patients, and an earlier detection and intervention must be needed to treat severe infection in these patients [97–99]. More data is required for an accurate perception of the outcomes and admission criteria to the ICU, in order to determine the best prognostic parameters [100, 101]. The generally accepted rules for hemodynamic support can be followed [102].

## 12. Conclusion

The ICU physician must be familiarized with the complex alterations in physiological systems related to LC. Cardiocirculatory dysfunction is present previous to the decompensating end-stage liver disease and is frequently unrecognized. CCM is a novel clinical condition that can aggravate the hemodynamic condition and/or support. Although there are no standard accepted diagnostic methods, echocardiography can be quite useful. Renal disease and pulmonary disease are also subject to special conditioning in LC. Diagnostic criteria for HPS and HRS must be thoroughly observed. Infectious problems in LC patients are probably the most life threatening. As the typical events for severe infection can occur more rapidly, a high detection level and prompt antibiotherapy must be considered.

Several liver support devices are available, and the ICU physician must be familiar with their use. All together, the current care for LC patients requires specific training dedicated personnel in a multidisciplinary team, in which the ICU physician plays a critical role. The connection with a liver transplantation center is, therefore, mandatory.

## Figures and Tables

**Table 1:** The pathophysiology of cardiovascular abnormalities.

Attenuated systolic and diastolic response to stress stimuli	

Structural and histological changes in cardiac chambers	

Electrophysiological abnormalities	

Increased serum markers of cardiac stress	

Altered function of *β*-adrenergic and muscarinic receptors:	
(i) Reduced receptor density in cirrhotic patients and animal model	
(ii) Enhanced muscarinic: contributing to the negative inotropic effect on the myocardium	
(iii) Altered membrane fluidity: these changes have a profound effect on the *β*- adrenoceptor function that includes impairing the receptor-ligand interaction and affects the function of membrane-bound ion channels (Ca^++^ and K^+^)	

NO, carbon monoxide, and endocannabinoids exerts a negative effect on cardiac contractility	

**Table 2:** Definition of cirrhotic cardiomyopathy.

Systolic dysfunction	(i) Blunted increase in cardiac output with stress (ii) Resting LVEF <55%

Diastolic dysfunction	(i) E/A ratio <1 (ii) Prolonged DT (>200 msec) (iii) Prolonged IVRT (>80 msec)

Supportive criteria	(i) Electrophysiological abnormalities (ii) Altered chronotropic response (iii) Electromechanical dyssynchrony (iv) Prolonged QTc (v) Enlarged LA (vi) Increased cardiac mass (vii) Increased BNP/proBNP (viii) Increased troponin I

**Table 3:** Stages of portopulmonary hypertension.

Stage	MPAP (mmHg)	Regarding liver transplantation
Mild	25–35	No added risk of perioperative morbidity or mortality
Moderate	35–44	High risk of perioperative morbidity and mortality; considering vasodilator therapy; relative contraindication for liver transplantation
Severe	>45	Absolute contraindication for liver transplantation

**Table 4:** Diagnosis of pulmonary hypertension.

Idiopathic pulmonary arterial hypertension	
Pulmonary arterial hypertension associated with: (i) Connective tissue diseases (ii) Congenital heart diseases (iii) Portopulmonary hypertension (iv) HIV infection	

Left heart disease: (i) Systolic and diastolic dysfunction (ii) Valvular heart disease	

Primary lung diseases and/or chronic hypoxia (i) Chronic obstructive pulmonary disease (ii) Interstitial lung disease (iii) Sleep-disordered breathing	

Chronic thromboembolism	

**Table 5:** Treatment options for hepatopulmonary syndrome.

Prostacyclin analogues	Potent pulmonary and systemic vasodilators Antagonist of platelet aggregation Epoprostenol is the most commonly used drug in this class, is efficacious, but has several adverse effects related to safety, tolerability, and route of administration (short half-life, requiring the insertion of central venous access) Nebulized iloprost, 6–9 times a day, has shown improved symptoms, exercise tolerance, and functional capacity Treprostinil, a stable and long-acting prostacyclin analogue, can be administered subcutaneously and intravenous

Endothelin antagonist	Bosentan, a nonselective ET-1 receptor antagonist, improves exercise tolerance, functional capacity, and pulmonary hemodynamics, but also can worsen hepatic dysfunction and deteriorate renal failure especially in patients with type 2 HRS Ambrisentan: a selective ET_A_ receptor antagonist, oral and once a day administration, with minimal hepatotoxicity risk

Phosphodiesterase-5-inhibitors	Sildenafil and tadalafil, their use results in an increased NO-mediated vasodilatation in the pulmonary vasculature

Vasopressin analogues	Terlipressin: several reports have shown that the administration of terlipressin decreases pulmonary artery pressure in cirrhotic patients with mild pulmonary hypertension, and reduces portal pressure, improves hyperdynamic circulation and functional renal failure via stimulation of mesenteric V1 receptors [12]

**Table 6:** Diagnostic criteria of hepatorenal syndrome.

Chronic or acute hepatic disease with advanced hepatic failure and portal hypertension.	
A plasma creatinine concentration above 1.5 mg/dL (133 micromol/L) that progresses over days to weeks.	
The absence of any other apparent cause for the renal disease, including shock, ongoing bacterial infection, current or recent treatment with nephrotoxic drugs, and the absence of ultrasonographic evidence of obstruction or parenchymal renal disease.	
Urine red cell excretion of less than 50 cells per high power field (when no urinary catheter is in place) and protein excretion less than 500 mg/day.	
Lack of improvement in renal function after volume expansion with intravenous albumin (1 g/kg of body weight per day up to 100 g/day) for at least two days and withdrawal of diuretics.	

**Table 7:** The West Haven criteria for hepatic encephalopathy.

Grade 0	Minimal hepatic encephalopathy, no asterixis, and trivial changes in personality or behavior
Grade 1	Trivial lacks of awareness, attention, sleep disturbances, altered mood, and asterixis
Grade 2	Lethargy, apathy, time disorientation, amnesia to recent events, inappropriate behavior, slurred speech, asterixis.
Grade 3	Somnolence, confusion, time and place disorientation, bizarre behaviour, clonus, nystagmus, positive Babinski sign, and absent asterixis
Grade 4	Coma

**Table 8:** Oral antibiotics in hepatic encephalopathy.

Neomycin	There are no well-designed studies Long-term use limited by nephrotoxicity and ototoxicity Chronic hepatic encephalopathy: 1–4 g/day Acute hepatic encephalopathy: 1–2 r every 4–6 hours

Metronidazole	Not approved for hepatic encephalopathy Side effects: neurotoxicity and gastrointestinal disturbances. Oral dose: 250 mg every 12 hours

Rifaximin	Multiple studies have demonstrated safety and efficacy for the treatment of acute and chronic encephalopathy Two trials compared rifamixine versus rifamixin with lactulose and lactitiol and suggested that rifamixin produces equal or superior improvement of ammonia levels and hepatic encephalopathy in shorter time
